# Septic Shock Secondary to Severe Gastroenteritis Resulting From Sapovirus Infection

**DOI:** 10.7759/cureus.24010

**Published:** 2022-04-10

**Authors:** Eric Landa, Saad Javaid, Jung S Won, Erika Vigandt, Jonathan Caronia, Parvez Mir, Zeyar Thet

**Affiliations:** 1 Internal Medicine, Unity Health, Searcy, USA; 2 Internal Medicine, Wyckoff Heights Medical Center, New York City, USA; 3 Internal Medicine, The Brooklyn Hospital Center, New York City, USA; 4 Pulmonary and Critical Care Medicine, Northwell Health, New York City, USA; 5 Pulmonary and Critical Care/Internal Medicine, Wyckoff Heights Medical Center, New York City, USA; 6 Internal Medicine and Infectious Diseases, Wyckoff Heights Medical Center, New York City, USA

**Keywords:** infectious disease, gastroenterology, sepsis, septic shock, sapovirus

## Abstract

Sapovirus causes acute gastroenteritis (AGE) which manifests as severe diarrhea and vomiting. It is most often seen in, but not limited to, children and toddlers but can occur in people of all ages. It is typically more prevalent in low to middle-income countries but has also been reported in progressive countries such as the United States. Due to the universal use of reverse transcriptase-polymerase chain reaction (RT-PCR) testing, the reported incidence of sapovirus has continued to grow as the culprit agent in both AGE outbreaks and isolated cases. Its symptoms resemble what is seen with rotavirus but with a milder clinical course. This discussion explores the dire implications of a relatively understated pathogen. Here, we present a rare case of a 20-year-old woman who presented with septic shock secondary to severe gastroenteritis as a result of sapovirus infection.

## Introduction

Sapovirus was initially reported in 1976 in human diarrheal samples by examination of the virus using microscopy [1]. Following this, a broad range of hosts has been recognized, including bats, pigs, sea lions, and dogs [2]. Even though early studies described sapovirus infections to be associated with less severe symptoms than norovirus and rotavirus, later studies have demonstrated that sapovirus infections can result in hospitalizations and severe dehydration [3]. The US studies have estimated sapovirus causes nine to 23 cases in the community and one to two outpatient visits per 1000 person-years, representing ~2% of all acute gastroenteritis [4]. Here, we present an unusual case of a young adult who presented with life-threatening septic shock requiring pressor support and care in the intensive care unit (ICU), caused by an infection with sapovirus.

## Case presentation

A 20-year-old female with no significant medical history presented to the emergency department (ED) complaining of multiple episodes of non-bloody, non-bilious vomiting and watery diarrhea for the past one day. She also reported subjective fevers, chills, and a headache. She denied any changes in her diet, dysuria, recent illness, travel, or sick contacts at home. In the ED, her vital signs were significant for temperature 99.0°F, blood pressure 84/46 mmHg, heart rate 115 beats per minute, respiratory rate 34 breaths per minute, and oxygen saturation 100%. She received 6 L of IV fluids to which her blood pressure did not respond to and peripheral norepinephrine was started for pressure support.

**Figure 1:**
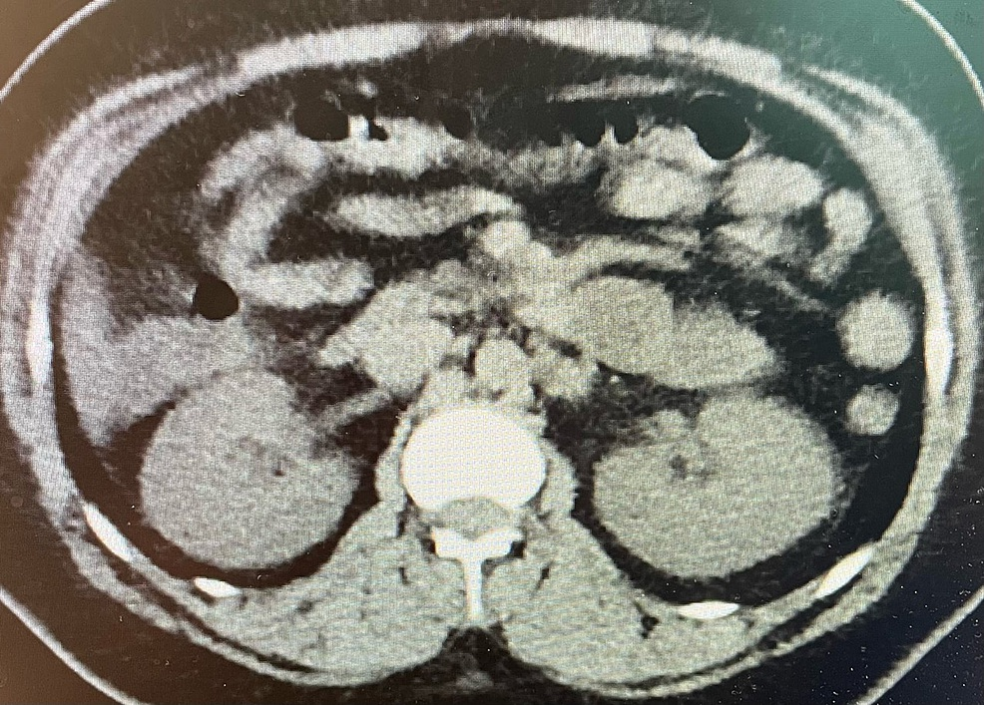
CT abdomen/pelvis with contrast demonstrating no acute pathology.

Her labs were significant for white blood cell count 26.0 109/L, lactic acid 4 mmol/L, BUN 43 mg/dL, Cr 3.80 mg/dL, D-dimer >10,000 ng/mL, and transaminitis - aspartate aminotransferase (AST) 158 U/L, alanine aminotransferase (ALT) 193 U/L. CT angiography of the chest was done, which ruled out a pulmonary embolism. A CT of the abdomen/pelvis with contrast was performed as well, revealing no acute pathology (Figure 1). Abdominal ultrasound demonstrated a fatty liver.

She was started on cefepime, metronidazole, and oral (PO) vancomycin due to suspicion of possible *Clostridioides difficile* infection. Coronavirus testing was performed and was negative two times. Blood cultures obtained showed no growth. A urinalysis was negative for a urinary tract infection however a urine culture grew Gram-negative rods susceptible to cefepime. *C. difficile *toxin came back negative and PO vancomycin was discontinued. Stool comprehensive profile PCR, which tests for bacterial and viral infections, was performed and came back positive for sapovirus, which was the only culprit capable of explaining her symptoms of acute severe gastroenteritis (Table 1).

**Table 1:** Stool comprehensive profile.

Stool comprehensive profile	Detection
Campylobacter	Not detected
*Clostridium difficile* toxin A/B	Not detected
Salmonella	Not detected
Vibrio cholera	Not detected
Yersinia enterocolitica	Not detected
Escherichia coli	Not detected
Shiga toxin	Not detected
Cryptosporidium	Not detected
Entamoeba histolytica	Not detected
Giardia duodenalis	Not detected
Adenovirus	Not detected
Astrovirus	Not detected
Norovirus	Not detected
Rotavirus	Not detected
Sapovirus	Detected

After a two-day ICU stay, she was weaned off pressor support and her vital signs normalized. She was transferred to the regular medicine floor where she continued to improve and was subsequently discharged home. Since then, she followed up at the medicine outpatient clinic in five days time and has been doing well, she no longer has had any abdominal pain, diarrhea, or other complaints and has made a full recovery.

## Discussion

Sapovirus belongs to the family of Caliciviridae. Unlike norovirus, which is genetically diverse and widely studied due to its high prevalence among all the diarrheal viruses detected, limited studies are available on sapoviruses (SaV). The majority of the sapovirus outbreak cases are observed in children and very few cases are reported in the adult population.

SaV is divided into five genogroups GI-GV. Each genogroup also has different genotypes. Genogroups I, II, IV, and V infect humans, whereas genogroup III infects porcine species [5]. Different studies have been conducted across the globe at different times, and it was observed that the predominant genotypes vary by region and geographic characteristics. SaV of genogroup I genotype 1 (GI 1) was the dominant strain in the United Kingdom from 1989 to 2004. While GII 1 was the predominant strain in 2004 [6]. Similarly, a study conducted in Japan from 2014 to 2017 showed that GI 1 was the predominant strain followed by GII 1, GIV 1, GI 2, GI 3, and GII 3 [7]. It is proposed that variation in sapovirus genotype combined with host susceptibility lead to the emergence of GIV in late 2006 and 2007, which were believed to be the causative agent in the outbreaks in long-term care facilities and senior lodges. Similarly, a potential linkage was observed between the sapovirus genotype evolution from GI/II to GIV and subsequent increased outbreak activity worldwide [8]. Infection with the sapovirus produces cellular and humoral immunity, though the mechanism remains partially understood. Reinfection with the same genotype is very uncommon, suggesting the development of protective immunity against the same genotype and also against the different genotypes within the same genogroup. However, the risk of infection from the other genogroups remains as no immunity is established against those genogroups.

The mechanism of diarrhea and vomiting associated with sapovirus infection is unclear. However, it is proposed that it causes decreased activity of brush border enzymes trehalase and alkaline phosphatase in the intestine, which leads to fat and D-xylose malabsorption causing diarrhea [9-11]. Gastric transit time and emptying are delayed, and it could lead to vomiting. However, the severity of vomiting is not related to the degree of emptying and is multifactorial in origin. Acute sapovirus infection causes reversible histopathological changes in the intestine; polymorphonuclear leukocytic infiltrations in the lamina propria with villous blunting and intact mucosa. The jejunum is primarily affected, sparring the stomach and rectum. These changes usually appear within 24 hrs of infection and can resolve in one to two weeks, although some changes have been present for more than six weeks [12-15]. Here we presented an atypical case of sapovirus infection in a young woman in her 20s resulting in septic shock requiring pressor support in the intensive care unit.

## Conclusions

With improved molecular recognition, sapovirus is now acknowledged as an important cause of diarrhea worldwide, mainly seen in young children. As no vaccines are presently available against sapovirus, primary prevention including good sanitation and safe food handling are key. More research is required to better understand transmission patterns and risk factors for sapovirus infection to guide prevention efforts. Treatment of sapovirus induced gastroenteritis is similar to diarrhea due to other viral causes and focuses on the use of oral rehydration. Here, we discussed an unusual case of severe gastroenteritis caused by sapovirus requiring ICU/critical level. We hope to bring light to this emerging viral infection and its ability to cause a life-threatening infection.
